# Modal Characterization of Glass–POE–Glass Laminate Specimens with Different Gel Contents for Non-Destructive Curing Assessment in Photovoltaic Applications

**DOI:** 10.3390/ma19204333

**Published:** 2026-10-15

**Authors:** Artūras Kilikevičius, Aleksandras Chlebnikovas, Egidijus Dragašius, Piotr Dubravskij, Algirdas Galdikas, Vytautas Makarskas

**Affiliations:** 1 Institute of Mechanical Science, Faculty of Mechanics, Vilnius Gediminas Technical University, Plytinės Str. 25, LT-10105 Vilnius, Lithuania; 2 Department of Production Engineering, Kaunas University of Technology, Studentų Str. 56, LT-51424 Kaunas, Lithuania; egidijus.dragasius@ktu.lt (E.D.); vytautas.makarskas@ktu.lt (V.M.); 3 Applied Research Institute for Prospective Technologies, Vismaliukų g. 34, LT-10243 Vilnius, Lithuania; piotr@metsolar.eu (P.D.); algis.galdikas@protechnology.lt (A.G.)

**Keywords:** polyolefin elastomer, photovoltaic encapsulant, gel content, crosslinking, modal analysis, frequency domain decomposition, non-destructive evaluation, interlayer shear coupling

## Abstract

The curing state of polyolefin elastomer (POE) encapsulants influences the mechanical behavior of laminated photovoltaic structures, while conventional gel-content (GC) determination requires destructive testing. This study evaluates vibration-based modal characterization as a potential non-destructive screening approach for distinguishing curing conditions in simplified glass–POE–glass laminates. Five GC levels (25%, 35%, 49%, 68%, and 75%) were investigated, with five independent laminate specimens tested at each level and five repeated modal measurements performed per specimen. The first six vibration modes were characterized using Frequency Domain Decomposition (FDD) and Enhanced Frequency Domain Decomposition (EFDD). The mean natural frequencies exhibited mode-dependent differences across the investigated curing conditions. Mode 1 varied by 2 Hz (1.34%), whereas Modes 2–6 showed peak-to-peak variations of 7–17 Hz (3.07–4.33%), with the largest relative variation observed for Mode 5. Modes 2–5 exhibited an apparent partial frequency recovery at GC = 75% following a decrease toward GC = 68%. However, the absence of retained individual frequency estimates prevented quantitative assessment of experimental variability and statistical confirmation of these differences. Finite element analysis supported modal correspondence, while a calibrated partial-composite-action model provided a structural interpretation of incomplete interlayer shear coupling. The results suggest potential for comparative non-destructive screening, but quantitative prediction of unknown GC requires further validation and uncertainty analysis.

## 1. Introduction

Photovoltaic (PV) modules are multilayer structures in which the encapsulant performs several functions simultaneously, including optical coupling, electrical insulation, mechanical protection, adhesion between adjacent layers, and resistance to moisture and environmental exposure. Consequently, the selection and processing of the encapsulant affect not only the electrical reliability and durability of a PV module but also the mechanical interaction between its constituent layers. This interaction is particularly relevant in glass–glass module architectures, where a relatively compliant polymeric interlayer transfers stresses between considerably stiffer glass layers [1,2,3,4].

Ethylene-vinyl acetate (EVA) has traditionally been one of the most widely used encapsulant materials in PV modules because of its optical transparency, processability, flexibility, and extensive industrial experience. However, increasing implementation of bifacial, glass–glass, and high-efficiency PV technologies has stimulated interest in alternative encapsulation materials. Polyolefin elastomers (POEs) have emerged as an important class of PV encapsulants because of their low polarity, favorable electrical insulation properties, reduced moisture permeability, and absence of acetate groups associated with acetic-acid formation in EVA-based systems. Recent research has consequently focused on the formulation, processing, crosslinking, mechanical performance, and long-term durability of POE encapsulants [5,6,7,8].

Commercial photovoltaic POE encapsulants are generally based on ethylene–α-olefin copolymers that undergo peroxide-initiated crosslinking during module lamination. The development of the polymer network depends on the molecular architecture of the encapsulant, crosslinking formulation, stabilizers and other additives, as well as on the thermal history imposed during lamination. Crosslinking restricts molecular-chain mobility and modifies rheological and mechanical characteristics of the material. Importantly, the curing behavior of POE cannot be assumed to be equivalent to that of EVA. Experimental dynamic mechanical analysis has demonstrated substantial differences between EVA and POE in crosslinking kinetics: for the investigated materials, POE exhibited slower gelation as well as a lower storage modulus and complex viscosity in the rubbery state [9,10,11].

Gel content (GC) is commonly used as an operational indicator of the crosslinking state of photovoltaic encapsulants. It represents the polymer fraction remaining insoluble after solvent extraction and therefore provides a practical measure associated with polymer-network formation. However, GC should not be interpreted as a direct measurement of Young’s modulus, shear modulus, or crosslink density. The measured value and its relationship with other curing indicators depend on the material formulation and the adopted analytical procedure. Recent investigations of commercial POE encapsulants have demonstrated that material-specific relationships between gel content and curing characteristics are required and that extraction and lamination procedures developed for other encapsulants cannot necessarily be transferred directly to POE [12,13].

The relationship between curing state and the mechanical behavior of POE is further influenced by polymer formulation and morphology. Modification of POE formulations can affect adhesion, creep resistance, thermal behavior, and other mechanical characteristics without producing equivalent changes in all material properties. Experimental studies of POE-based photovoltaic encapsulants have demonstrated that changes in formulation and lamination affect their mechanical, rheological, and adhesive behavior. Consequently, gel content should be regarded as an indicator of curing state rather than as a universal surrogate for the mechanical properties of a POE interlayer [14,15,16,17].

This distinction becomes particularly important when POE forms the compliant interlayer of a glass–POE–glass laminate. From a structural-mechanics perspective, such a system consists of stiff glass layers coupled through a substantially softer polymeric interlayer. The effective bending response therefore depends not only on the elastic properties of the individual layers but also on the efficiency of shear transfer through the polymer. Studies of laminated glass structures have shown that viscoelastic polymer interlayers exhibit frequency- and temperature-dependent behavior and that this behavior directly influences modal characteristics of the laminate. Accordingly, the dynamic stiffness of a glass–polymer–glass structure can occupy an intermediate state between the limiting cases of essentially uncoupled glass layers and fully composite behavior [18,19,20].

Experimental modal investigations further demonstrate that the polymer interlayer can measurably influence the vibration behavior of laminated glass structures. Hála et al. experimentally investigated large laminated-glass panels containing EVA or PVB interlayers and showed that interlayer type affects their oscillatory response, including natural frequencies and damping characteristics. These observations support the hypothesis that changes in the effective mechanical state of a polymer interlayer may be reflected in the modal response of the complete laminate [21,22,23].

Conventional evaluation of photovoltaic encapsulant curing is predominantly based on material-level analytical methods. Solvent extraction is used to determine the insoluble gel fraction, whereas differential scanning calorimetry and dynamic mechanical or rheological techniques can provide complementary information on residual curing reactions and the development of viscoelastic properties. For POE in particular, recent research has demonstrated correlations between gel-content measurements and calorimetry-based curing indicators while emphasizing that these relationships remain material-specific [9,12].

Spectroscopic techniques represent another potential route for evaluating changes introduced during lamination. A recent study of EVA and POE photovoltaic encapsulants investigated changes in infrared absorption-band characteristics and reported relationships between selected spectral features, polymer transformations, and gel content after thermal lamination. Such approaches demonstrate growing interest in developing alternative methods for encapsulant-quality assessment. Nevertheless, spectroscopy primarily provides information about the polymer material itself, whereas vibration-based characterization can potentially evaluate the integrated mechanical response of the complete laminated structure [24].

Modal characterization provides a complementary structural-level perspective because natural frequencies, damping characteristics, and mode shapes are governed by the effective distribution of mass, stiffness, energy dissipation, boundary conditions, and internal mechanical coupling. Experimental modal studies of laminated glass have demonstrated that the polymer interlayer affects the vibration response, including natural frequencies and damping characteristics, thereby linking interlayer mechanical behavior to the dynamic performance of the complete laminate [21,22,23]. More generally, recent investigations of plate-like composite structures have demonstrated the sensitivity of dynamic wave characteristics to changes in layered structural configurations, further supporting the use of vibration- and wave-based approaches for non-destructive structural assessment [25]. The relevance of modal characterization to photovoltaic structures has also been demonstrated by experimental and finite element investigations of conventional glass-based and lightweight composite PV modules [26]. These investigations showed that module construction and the mechanical contribution of individual constituent layers affect modal frequencies and mode shapes. Such modal characteristics are relevant to application performance because dynamic loading can excite structural resonances, while changes in laminate stiffness, mass distribution, or interlayer coupling can alter the vibration response. Modal characterization can therefore provide both information relevant to structural dynamic performance and a non-destructive means of detecting changes in the effective mechanical state of layered PV structures [26].

Frequency Domain Decomposition (FDD) and Enhanced Frequency Domain Decomposition (EFDD) provide response-based approaches for identifying structural modal parameters from measured vibration signals. In the present study, repeated impact events are used to generate broadband structural excitation, while the measured excitation force is not included in the modal identification procedure. Natural frequencies and mode shapes are identified from acceleration responses using FDD, whereas EFDD is additionally applied to estimate damping characteristics. The procedure is therefore treated herein as output-only modal identification under broadband impact excitation.

Despite substantial research on POE formulation, crosslinking kinetics, lamination, durability, adhesion, and material-level characterization, the relationship between the curing state of a POE interlayer and the modal response of a glass–POE–glass laminate relevant to photovoltaic applications remains insufficiently investigated. Existing POE studies have primarily addressed chemical, thermal, rheological, spectroscopic, or durability-related indicators of encapsulant properties, whereas the possibility of assessing curing-induced changes through the integrated structural dynamic response of a laminated system has received considerably less attention [9,12,24].

Furthermore, because gel content does not uniquely define the mechanical and viscoelastic properties of POE, a simple monotonic relationship between GC and the modal characteristics of a laminated structure cannot be assumed a priori. The vibration response is expected to reflect the combined structural effect of the polymer interlayer and its ability to transfer shear between adjacent glass layers rather than gel content as an isolated material parameter [12,18].

The objective of the present study is therefore to investigate the sensitivity of the modal characteristics of glass–POE–glass laminate specimens with different POE curing states characterized by gel contents of 25%, 35%, 49%, 68%, and 75%. The first six vibration modes are identified experimentally using FDD and EFDD. The measured structural response is subsequently interpreted using finite element analysis and a calibrated partial-composite-action model representing incomplete shear interaction between the glass layers. Statistical relationships between GC and the identified natural frequencies are additionally examined to evaluate their relative sensitivity to changes in curing state. The principal contribution of this study is the evaluation of the integrated structural dynamic response of a glass–POE–glass laminate relevant to photovoltaic applications as a potential non-destructive indicator of encapsulant curing state. At the present stage, the proposed approach is considered a screening methodology for differentiating curing states rather than a fully validated method for quantitative prediction of unknown gel content.

## 2. Materials and Methods

### 2.1. Test Specimens and Sample Preparation

Glass–POE–glass laminated specimens without photovoltaic cells were manufactured to investigate the influence of the curing state of the polyolefin elastomer (POE) interlayer on the dynamic response of the laminate. Five curing conditions were considered, corresponding to experimentally determined POE gel-content levels of 25%, 35%, 49%, 68%, and 75%. For each curing condition, five independent glass–POE–glass laminate specimens were manufactured for experimental modal testing, resulting in a total of 25 laminate specimens. Each specimen was subjected to five repeated modal measurements.

Silicon photovoltaic cells were intentionally excluded from the specimens in order to isolate the mechanical contribution of the encapsulant and the glass–POE–glass interaction. This simplified laminate configuration eliminates additional local stiffness and mass variations associated with silicon cells, metallization, interconnects, and cell-to-encapsulant interfaces, thereby allowing changes in modal characteristics to be related more directly to the mechanical state of the POE interlayer.

Each specimen consisted of two glass layers separated by a POE encapsulant layer. The glass layers were manufactured from Guardian UltraClear low-iron soda–lime–silicate float glass (Guardian Industries, Auburn Hills, MI, USA), with a nominal thickness of 3.6 mm produced to a special order. The glass type was identified from the manufacturer’s technical documentation, which specifies compliance with EN 572-1 and EN 572-2 for soda–lime–silicate float glass. The nominal material properties and layer thicknesses adopted for structural characterization and numerical modelling are summarized in Table 1. The planar dimensions of the laminated specimens were approximately 415 × 297 mm.

Lamination was performed using an ICOLAM 18/11 laboratory laminator (Meier Solar Solutions, Bocholt, Germany). The laminate stacks were manually assembled before being introduced into the preheated lamination chamber. The lamination temperature was maintained at 150 °C for all investigated specimens. After placement in the laminator, the chamber was evacuated to approximately 0.1 mbar. Following a vacuum stage of 340 s, a silicone membrane was applied at a pressure of approximately 800 mbar. Different curing states were produced by varying the duration of the crosslinking stage while maintaining the remaining lamination parameters unchanged.

Separate laminate preparations intended for gel-content determination incorporated thin polytetrafluoroethylene (PTFE) separation films between the POE and adjacent glass surfaces. The PTFE layers facilitated removal of the cured POE after lamination without mechanically damaging or contaminating the polymer material. For each investigated curing condition, the PTFE-containing preparations and the corresponding glass–POE–glass specimens intended for modal testing were processed within the same lamination cycle, using the same temperature, pressure, vacuum, and curing-time settings. The PTFE-containing preparations were used exclusively for subsequent gel-content determination and were not used for modal testing. Thus, the GC values were assigned to the modal specimens on the basis of their shared lamination conditions rather than determined directly from the modal specimens themselves.

The resulting curing conditions produced the five POE gel-content levels investigated in the present study. In the following analysis, GC is therefore used as an experimental descriptor of the POE curing state rather than as a direct measure of the elastic or shear modulus of the encapsulant.

### 2.2. Gel Content Determination

The curing state of the POE encapsulant was characterized by its gel content (GC), determined using solvent extraction. POE samples intended for GC analysis were separated from the glass surfaces after lamination using the PTFE release layers described above. Five independent gel-content determinations were performed for each investigated curing condition, and the arithmetic mean of these determinations was used to assign the corresponding GC level (25%, 35%, 49%, 68%, or 75%) to the modal-test specimens manufactured under the same lamination conditions.

Approximately 1 g of POE material was collected for each determination and cut into pieces of approximately 10 × 10 mm^2^. The initial dry mass, m_0_, was measured using a precision balance before extraction. The polymer specimens were subsequently subjected to extraction in xylene (Reag. Ph. Eur., CARLO ERBA Reagents S.A.S., Val-de-Reuil, France) at 110 ± 1 °C for at least 12 h.

Following extraction, the solvent was removed and the remaining insoluble polymer fraction was dried at 110 ± 1 °C for at least 8 h to remove residual solvent. After drying, the specimens were weighed again to determine the mass of the insoluble fraction, m_g_.

Gel content was calculated as:

(1) $$\mathrm{GC}=\frac{m_{g}}{m_{0}}\times 100\%,$$

where m_0_ is the initial dry mass of the POE specimen before extraction and m_g_ is the dry mass remaining after solvent extraction.

The measured gel content represents the solvent-insoluble fraction of the POE and is used in this study as an operational indicator of the curing state. It is not interpreted as a direct measurement of crosslink density or elastic modulus. The investigated laminates were classified according to five GC levels: 25%, 35%, 49%, 68%, and 75%.

### 2.3. Experimental Modal Testing

Vibration measurements were performed to characterize the dynamic response of the glass–POE–glass laminates at the investigated curing states. Five piezoelectric accelerometers were mounted at predefined measurement locations on each specimen and connected to a Brüel & Kjær LAN-XI data acquisition system. The same sensor configuration and measurement procedure were maintained for all specimens to ensure consistency between the investigated GC levels.

During testing, each specimen was placed horizontally on a compliant foam support in order to approximate free–free boundary conditions and minimize the influence of the supporting structure on the measured modal parameters.

Broadband structural excitation was generated using an instrumented impact hammer. Although the excitation force was measured by the instrumented hammer, the force signal was not included in the subsequent modal identification. Only the acceleration responses measured by the five accelerometers were used for modal parameter estimation. Five repeated modal measurements were performed on each of the five independently manufactured laminate specimens at every GC level. Thus, the experimental campaign comprised 25 physical laminate specimens and 125 repeated modal measurements across the five curing conditions. The repeated measurements were conducted using the same predefined accelerometer locations, measurement procedure, and compliant-support arrangement to reduce test-configuration variability. However, individual measurement-level modal-frequency estimates and their dispersion are not available in the retained dataset, and the separate contributions of impact-to-impact variability, support-condition variability, and sensor mounting/repositioning uncertainty cannot be quantified retrospectively. Accordingly, the reported modal-frequency differences are interpreted without a formal experimental uncertainty estimate.

**Figure 1:**
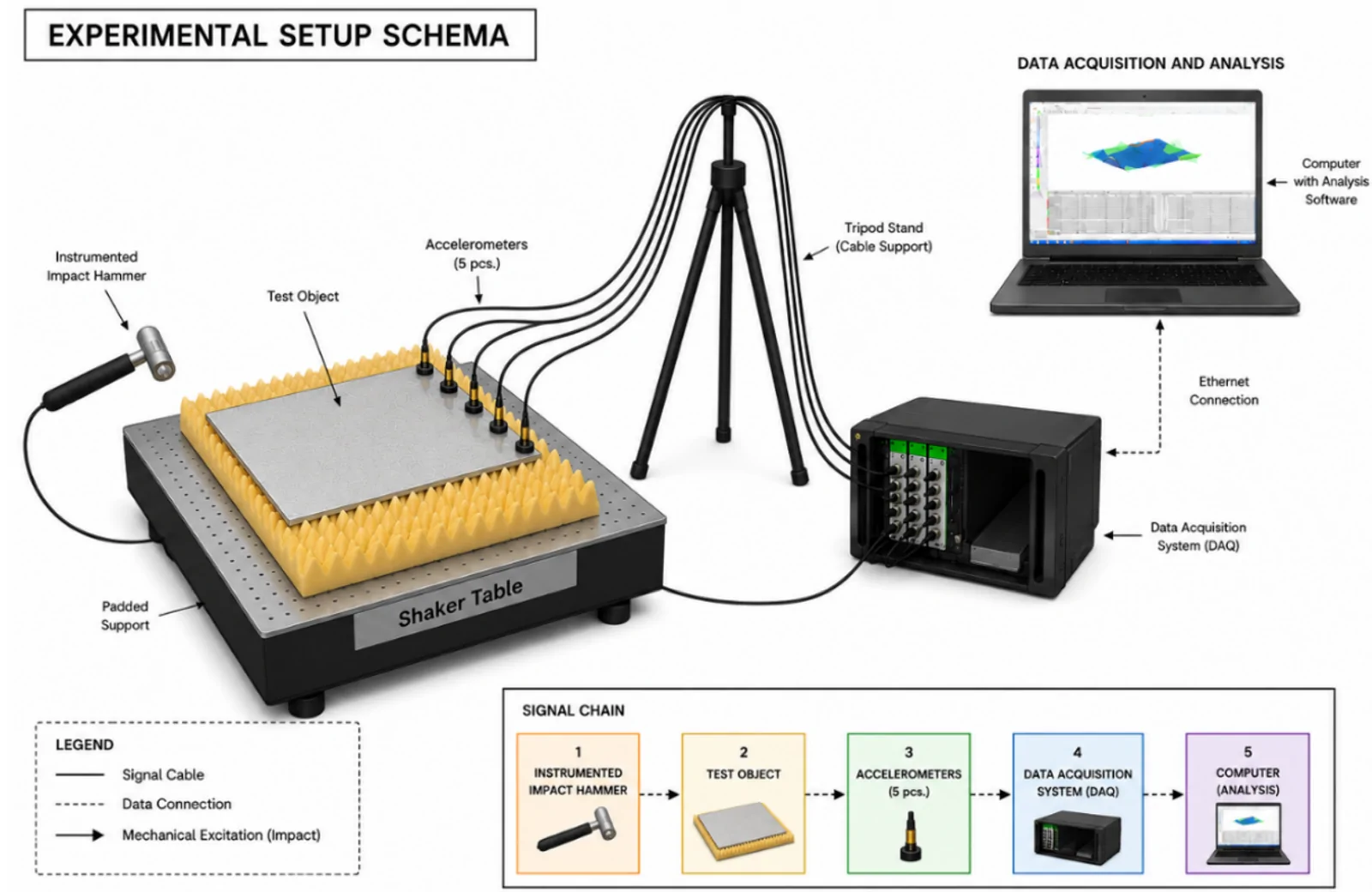
Schematic representation of the experimental modal-testing configuration, including the glass–POE–glass specimen, impact excitation, five accelerometers, LAN-XI data acquisition system, and BK Connect processing environment.

**Figure 2:**
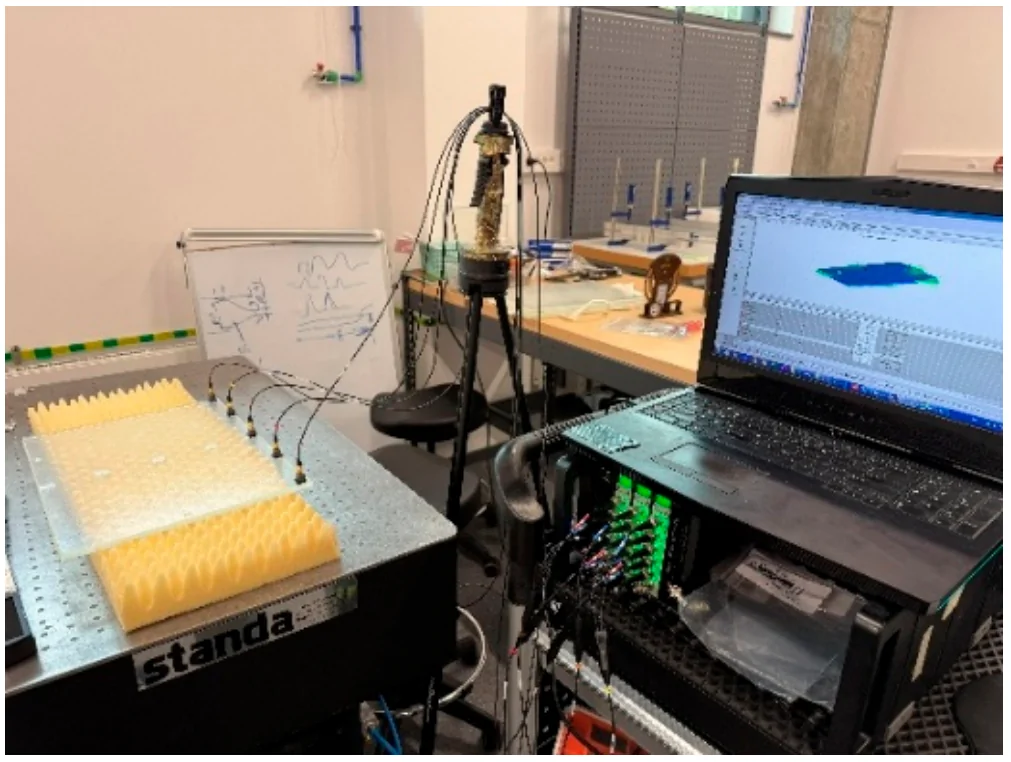
Actual laboratory arrangement used for vibration measurements of the glass–POE–glass laminated specimens, showing the compliant foam support employed to approximate free–free boundary conditions.

**Figure 3:**
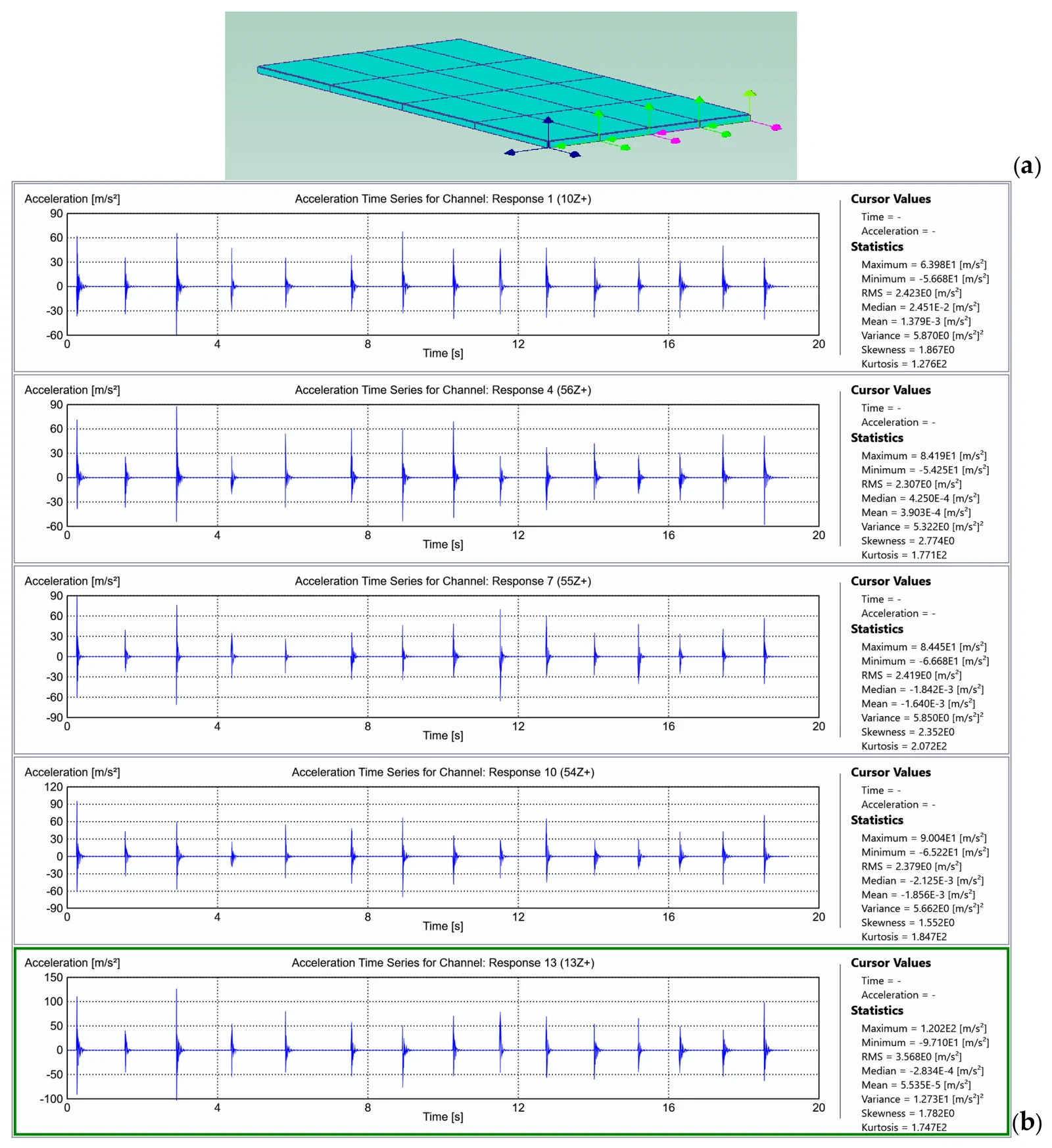
Experimental response measurements for the GC = 68% specimen: (**a**) accelerometer measurement locations on the glass–POE–glass laminate; (**b**) representative acceleration time histories recorded at the five measurement locations during repeated impact excitation.

Data acquisition and signal processing were performed using Brüel & Kjær BK Connect software (version 2025.1, Brüel & Kjær, Naerum, Denmark). The experimental configuration, including the specimen, accelerometer locations, impact excitation, LAN-XI acquisition system, and data-processing environment, is schematically presented in Figure 1. Figure 1 was prepared with the assistance of ChatGPT (OpenAI, San Francisco, CA, USA, GPT-5.6) to improve the visual clarity and graphical representation of the experimental equipment. The AI-assisted graphical output was carefully reviewed and verified by the authors to ensure its accuracy and consistency with the actual experimental setup and the methodology described in this study. Figure 2 documents the actual laboratory test arrangement, including the compliant foam support used to approximate free–free boundary conditions, and is provided to clarify the physical support conditions applied during the modal measurements. Representative acceleration time histories measured at the five sensor locations for the GC = 68% specimen are presented in Figure 3.

### 2.4. Modal Parameter Identification Using FDD and EFDD

Modal parameters were identified from the measured acceleration responses using Frequency Domain Decomposition (FDD) and Enhanced Frequency Domain Decomposition (EFDD). Although an instrumented hammer was used and the excitation force was recorded, the present analysis was intentionally formulated as response-based output-only modal identification. The hammer impacts served as broadband excitation events, whereas modal frequencies and mode shapes were extracted consistently from the spectral density matrix of the measured acceleration responses. This approach was selected to evaluate whether curing state-related differences could be identified from structural response measurements without requiring the excitation force signal as an input to the identification procedure. Accordingly, the measured impact-force signal was not used to construct frequency-response functions (FRFs) or to perform conventional input–output experimental modal analysis. A direct comparison between the present output-only procedure and force-normalized FRF-based modal identification was not performed and remains a subject for future validation.

Let the vector of simultaneously measured acceleration responses be denoted by:

(2) $$y\left(t\right)={\left[y_{1}(t)\;y_{2}(t)\;y_{1}(t)\ldots y_{m}(t)\right]}^{T},$$

where *y*(*t*) is the vector of simultaneously measured acceleration responses, *y_m_*(*t*) is the acceleration response of the *m*-th measurement channel, *t* denotes time, *m* = 5 is the number of measurement channels, and the superscript *T* denotes the transpose. In the frequency domain, the measured responses were represented by the spectral-density matrix:

(3) *G_yy_*(*f*),

where *G_yy_*(*f*) is the response spectral-density matrix, *f* denotes frequency, and its elements contain the auto- and cross-spectral densities between the acceleration channels. For each discrete frequency f, singular value decomposition of the response spectral-density matrix was performed as:

(4) *G_yy_*(*f*) = *U*(*f*)Σ(*f*)*U^H^*(*f*),

where *U*(*f*) contains the singular vectors, Σ(*f*) is the diagonal matrix of singular values, and the superscript *H* denotes the conjugate transpose.

Structural resonances were identified from peaks in the dominant singular-value spectra. At frequencies dominated by an individual structural mode, the corresponding singular vector provides an estimate of the mode-shape vector. Modal frequencies and mode shapes were consequently identified using FDD from the singular-value spectra of the measured acceleration responses.

EFDD was additionally employed to obtain refined estimates of the modal frequencies and damping ratios. For each identified mode, the spectral region associated with the corresponding singular-value peak was isolated and transformed into the time domain. The resulting modal correlation function was used to estimate the damped oscillation characteristics and modal damping.

The first six elastic vibration modes were retained for subsequent comparison between the investigated GC levels. Mode correspondence between specimens was established from the identified modal frequencies and deformation patterns so that equivalent structural modes were compared throughout the study.

The FDD frequencies were subsequently used as the primary modal indicators for evaluating the relationship between POE gel content and structural dynamic response. EFDD results were used to support modal identification and to characterize damping for the reference GC = 68% specimen.

### 2.5. Finite Element Model

A three-dimensional finite element (FE) model of the glass–POE–glass laminate was developed in COMSOL Multiphysics 6.3 to support the interpretation of the experimentally identified vibration modes and to compare the measured and numerically predicted natural frequencies. The numerical analysis was performed for the reference laminate corresponding to GC = 68%. The FE model was not intended to reproduce the experimentally observed frequency–GC relationship; rather, it was used as a reference for evaluating modal-frequency and mode-shape correspondence.

The model reproduced the geometry of the experimental glass–POE–glass laminate. The material properties adopted in the numerical analysis are summarized in Table 1. The glass and POE layers were represented as homogeneous, isotropic, linear-elastic materials. For the reference GC = 68% specimen, an effective POE Young’s modulus of 11 MPa was adopted.

Perfect mechanical continuity was assumed between adjacent glass and POE layers. Accordingly, relative separation and sliding at the glass–POE interfaces were neglected. This assumption represents an idealized perfectly bonded interface and allows mechanical interaction between the glass layers through deformation of the comparatively compliant POE interlayer.

**Figure 4:**
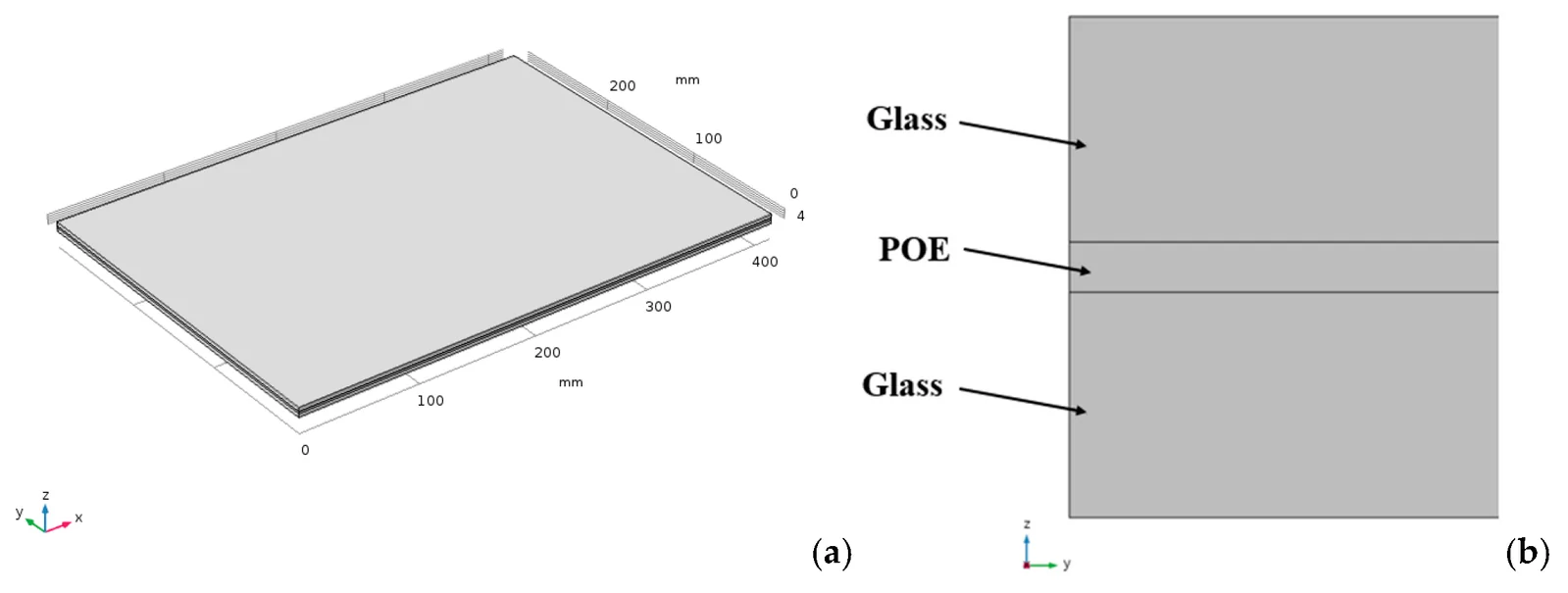
Finite element representation of the glass–POE–glass laminate in COMSOL Multiphysics: (**a**) general model view; (**b**) layered laminate configuration.

**Figure 5:**
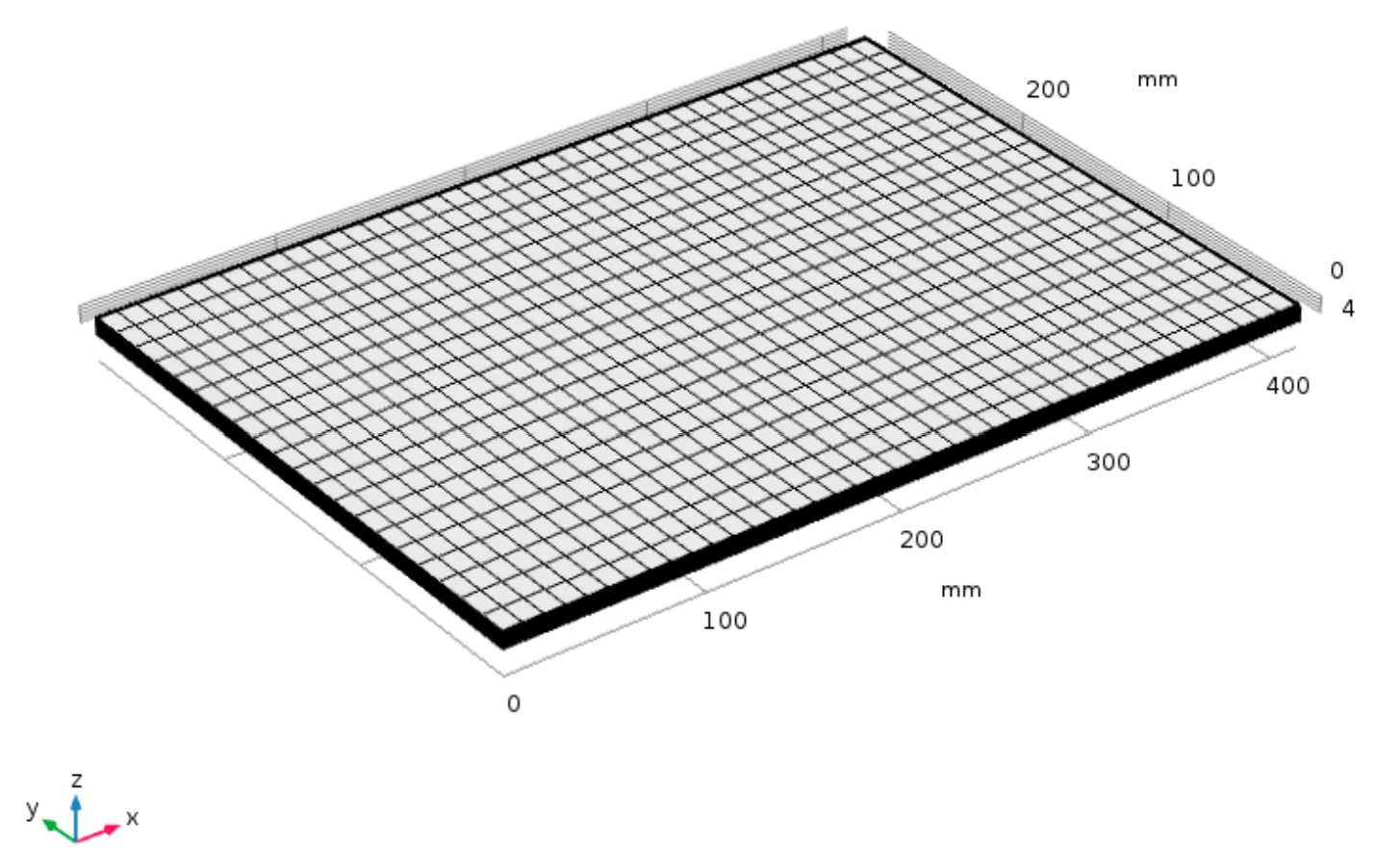
Finite element discretization of the glass–POE–glass laminate.

The FE discretization consisted of approximately 8800 finite elements connected through 10,332 nodes. The numerical representation of the laminate and its discretization are shown in Figure 4 and Figure 5, respectively.

The experimental support arrangement was approximated using free–free boundary conditions. The rigid-body modes associated with unconstrained motion were excluded from the comparison, and the first six elastic structural modes corresponding to the experimentally identified vibration modes were considered.

The natural frequencies and corresponding mode shapes were obtained from linear eigenfrequency analysis. For an undamped system, the governing eigenvalue problem can be expressed as:

(5) $$\left(K-\omega_{i}^{2}M\right)\varphi_{i}=0,$$

where K and M denote the global stiffness and mass matrices, respectively, ω*_i_* is the angular natural frequency in rad/s of the *i*-th mode, and φ_i_ is the corresponding eigenvector.

The natural frequency in Hz was calculated as:

(6) $$f_{i}=\frac{\omega_{i}}{2\pi },$$

where *f_i_* is the natural frequency in Hz of the *i*-th vibration mode.

The first six calculated elastic mode shapes and natural frequencies were subsequently compared with the experimentally identified FDD results for the *GC* = 68% reference specimen. Because only one gel-content level was represented in the FE analysis, the numerical model was not used to establish or validate a functional relationship between POE gel content and natural frequency.

The FE representation involves several simplifying assumptions. The glass and POE layers were treated as homogeneous, isotropic, linear-elastic materials, with the POE interlayer represented by a constant effective Young’s modulus of 11 MPa for the *GC* = 68% reference specimen. The glass–POE interfaces were assumed to be perfectly bonded, with no relative separation or sliding between adjacent layers. The experimental foam-supported configuration was idealized using free–free boundary conditions, and structural damping was neglected in the linear eigenfrequency analysis. Temperature dependence, frequency dependence, viscoelasticity, and curing-dependent variation in the POE mechanical properties were not explicitly represented. Consequently, the FE model is used primarily to support modal correspondence and the structural interpretation of the experimentally identified response of the reference laminate rather than as a complete constitutive representation of the POE interlayer or as a predictive model of the frequency–*GC* relationship.

### 2.6. Calibrated Partial-Composite-Action Model

A simplified analytical model was employed to provide a complementary structural interpretation of the experimentally observed modal response. The glass–POE–glass specimen was represented as a laminated plate consisting of two stiff glass layers coupled through a comparatively compliant POE interlayer. Because the stiffness of the POE layer is substantially lower than that of glass, the structural response was assumed to lie between two limiting conditions: completely uncoupled glass layers and full composite action.

To represent this intermediate state, an effective bending stiffness was defined as:

(7) *D*_eff_ = *D*_uncoupled_ + *η*(*D*_full_ − *D*_uncoupled_),

(8) *η* = 0 (completely uncoupled glass layers),

(9) *η* = 1 (perfect composite action)

where *D*_eff_ is the effective bending stiffness of the laminate, *D*_uncoupled_ is the bending stiffness corresponding to completely uncoupled glass layers, *D*_full_ is the bending stiffness corresponding to full composite action, and η is a dimensionless partial-composite-action coefficient.

The coefficient η represents the effective structural interaction between the two glass layers provided by the POE interlayer. A value of η = 0 corresponds to the uncoupled limiting case, whereas η = 1 corresponds to full composite action. Intermediate values represent partial shear interaction. The coefficient is therefore treated as an effective structural calibration parameter and not as an intrinsic material property of POE.

The geometrical and material parameters adopted in the analytical model are summarized in Table 2.

The POE Young’s modulus range of 3–12 MPa was considered in the analytical representation, whereas the FE reference model employed the specific effective value of 11 MPa listed in Table 1.

The areal mass of the laminated specimen was calculated as:

(10) *m_A_* = *ρ_g_h_g_*_,total_ + *ρ*_POE_*h*_POE_, *m_A_* = 2500 × 0.0072 + 857 × 0.0008,
*m_A_* = 18.686 kg/m^2^, *m* = *m_A_ab* = 18.686 × 0.415 × 0.298 ≈ 2.31 kg.

For the completely uncoupled limiting case, in which the two glass layers deform independently and the POE interlayer transfers negligible shear forces, the bending stiffness was calculated as:

(11) $$D_{g}=\frac{E_{g}h_{g}^{3}}{12(1-\nu_{g}^{2})},\;D_{\mathrm{uncoupled}}=2\frac{E_{g}h_{g}^{3}}{12(1-\nu_{g}^{2})},\;D_{\mathrm{uncoupled}}=2\frac{70\times 10^{9}{\left(0.0036\right)}^{3}}{12\left(1-0.23^{2}\right)},\;D_{\mathrm{uncoupled}}\approx 574.72\;N\;m.$$

For the full-composite limiting case, perfect shear transfer through the POE interlayer was assumed such that both glass layers act as a single composite section. The distance from the neutral axis to the centroid of each glass layer was calculated as:

(12) $$z=\frac{h_{\mathrm{POE}}}{2}+\frac{h_{g}}{2},\;z=0.0004+0.0018=0.0022\;m.$$

(13) $$D_{\mathrm{full}}=2\frac{E_{g}}{1-\nu_{g}^{2}}\left(\frac{h_{g}^{3}}{12}+h_{g}z^{2}\right);\;D_{\mathrm{full}}=2\frac{70\times 10^{9}}{1-0.23^{2}}\left(\frac{{\left(0.0036\right)}^{3}}{12}+0.0036{\left(0.0022\right)}^{2}\right)\;D_{\mathrm{full}}\approx 3150.33\;N\;m\;574.72\leq D_{\mathrm{eff}}\leq 3150.33\;N\;m.$$

In Equations (10)–(13), m_A_ is the areal mass of the laminate, *ρ_g_* and *ρ*_POE_ are the densities of glass and POE, respectively, h_g,total_ and h_POE_ are the total glass thickness and POE interlayer thickness, respectively, *m* is the total laminate mass, *a* and *b* are the specimen length and width, *D_g_* is the bending stiffness of an individual glass layer, *E_g_*, *ν_g_*, and *h_g_* are the Young’s modulus, Poisson’s ratio, and thickness of an individual glass layer, respectively, and *z* is the distance from the laminate neutral axis to the centroid of each glass layer. *D_uncoupled_*, *D_full_*, and *D_eff_* denote the uncoupled, full-composite, and effective laminate bending stiffnesses, respectively.

Calibrated effective bending stiffness. The partial-composite-action coefficient η was calibrated against the experimentally identified first natural frequency. Using Equation (7), the effective bending stiffness was subsequently evaluated from the calibrated value of η. The resulting analytical model was used to provide an effective-stiffness interpretation of the experimentally observed first-mode response rather than as an independently validated predictor of the complete modal spectrum.

In the present analytical formulation, a single calibrated η range was used to represent the average partial composite interaction under the investigated experimental conditions. Consequently, the model was not intended to reproduce the detailed variation in modal frequencies with gel content. Instead, it provides a baseline representation of the global dynamic stiffness of the glass–POE–glass laminate. An extended GC-dependent formulation would therefore be required to explicitly represent the experimentally observed non-monotonic frequency–GC relationship.

Rayleigh–Ritz formulation for a free–free rectangular plate. During the experimental modal analysis, the specimens were placed on soft foam without mechanical clamping. Therefore, the boundary conditions were approximated as free–free. The transverse displacement was represented by a Rayleigh–Ritz series expansion. The corresponding strain and kinetic energies were formulated for the equivalent plate, and the stationary condition of the total energy led to the generalized eigenvalue problem.

(14) *w*(*x*,*y*,*t*) = *W*(*x*,*y*)*e^iωt^*;

(15) $$W\left(x,y\right)=\sum_{i}\sum_{j}q_{\mathrm{ij}}X_{i}\left(x\right)Y_{j}\left(y\right);$$

(16) $$U=\frac{D_{\mathrm{eff}}}{2}\iint_{A}\left[W_{,\mathrm{xx}}^{2}+W_{,\mathrm{yy}}^{2}+2\nu_{g}W_{,\mathrm{xx}}W_{,\mathrm{yy}}+2\left(1-\nu_{g}\right)W_{,\mathrm{xy}}^{2}\right]\;\mathrm{dA};$$

(17) $$T=\frac{\omega^{2}}{2}\iint_{A}m_{A}W^{2}\;\mathrm{dA};$$

(18) $$\frac{\partial \left(U-T\right)}{\partial q_{\mathrm{ij}}}=0;$$

(19) (K − *ω*^2^M)q = 0;

(20) $$f_{n}=\frac{\omega_{n}}{2\pi }.$$

In Equations (14)–(20), *w*(*x*,*y*,*t*) is the transverse displacement of the plate, *W*(*x*,*y*) is the spatial displacement function, *x* and *y* are the in-plane coordinates, *t* is time, $i=\sqrt{-1}$ is the imaginary unit, *ω* is the angular frequency, *q_ij_* are the Rayleigh–Ritz generalized coordinates, and *X_i_* (*x*) and *Y_j_* (*y*) are the assumed basis functions in the *x*- and *y*-directions, respectively. *U* and *T* denote the strain and kinetic energies, respectively; *D_eff_* is the effective bending stiffness, *ν_g_* is the glass Poisson’s ratio, *m_A_* is the laminate areal mass, and *dA* is the differential plate area. The terms *W*,*_xx_*, *W*,*_yy_*, and *W*,*_xy_* denote the corresponding second-order spatial derivatives of *W*(*x*,*y*). Furthermore, K and M are the Rayleigh–Ritz stiffness and mass matrices, respectively, *q* is the vector of generalized coordinates, *ω_n_* is the angular natural frequency of the *n*-th elastic mode, and *f_n_* is the corresponding natural frequency in Hz.

Because the plate is free in space, rigid-body translation and rotation modes have zero or near-zero frequencies and must be excluded from the set of elastic vibration modes.

The partial-composite-action coefficient introduced in the present study was not adopted directly from the literature. The underlying concept follows shear-coupled and effective-stiffness descriptions of laminated glass, whereas η was identified specifically from the experimentally measured modal response of the investigated glass–POE–glass laminate. Accordingly, η should be interpreted as an effective structural calibration parameter rather than as an intrinsic POE material property.

### 2.7. Statistical Analysis

The relationship between POE gel content and the experimentally identified natural frequencies was evaluated separately for each of the first six vibration modes. Because only five discrete GC levels (25%, 35%, 49%, 68%, and 75%) were investigated, the statistical analysis was treated as exploratory and was used primarily to characterize trends and compare the relative sensitivity of individual modes to changes in curing state.

Pearson’s correlation coefficient (r) was calculated for each vibration mode to quantify the strength and direction of the linear association between gel content and natural frequency. Because the experimentally observed frequency–*GC* relationship was not strictly monotonic, the linear correlation coefficient was not used as the sole measure of association.

To characterize the observed non-linear trend, a second-order polynomial regression was additionally fitted separately for each vibration mode:

(21) *f*(*GC*) = *a*∙*GC*^2^ + *b*∙*GC* + *c*,

where *f* is the experimentally identified natural frequency, *GC* is the gel content expressed in %, and *a*, *b*, and *c* are the fitted regression coefficients. The goodness of fit of each quadratic model was evaluated using the coefficient of determination (R^2^).

The absolute agreement between fitted and experimentally identified frequencies was additionally characterized using the root mean square error (RMSE) and mean absolute error (MAE):

(22) $$\mathrm{RMSE}=\sqrt{\frac{1}{n}\sum {\left(f_{\exp,i}-f_{\mathrm{fit},i}\right)}^{2}},$$

(23) $$\mathrm{MAE}=\frac{1}{n}\sum \left|f_{\exp,i}-f_{\mathrm{fit},i}\right|,$$

where *f_exp,i_* and *f_fit,i_* are the experimentally identified and fitted natural frequencies for the *i*-th *GC* level, respectively, *i* is the index of the investigated *GC* level, and *n* is the total number of investigated *GC* levels (*n* = 5).

Given the limited number of *GC* levels, no inferential significance testing was used to establish a statistically validated predictive relationship between gel content and natural frequency. Accordingly, Pearson’s r, R^2^, RMSE, and MAE were interpreted as descriptive measures of the observed frequency–*GC* trends rather than as evidence of a generalized predictive relationship.

## 3. Results

### 3.1. Experimental Modal Identification

The first six elastic vibration modes were consistently identified from the measured acceleration responses using FDD. EFDD was additionally applied to refine the modal-frequency estimates and to determine the corresponding damping ratios for the GC = 68% reference specimen. The modal parameters obtained for this specimen are summarized in Table 3.

**Figure 6:**
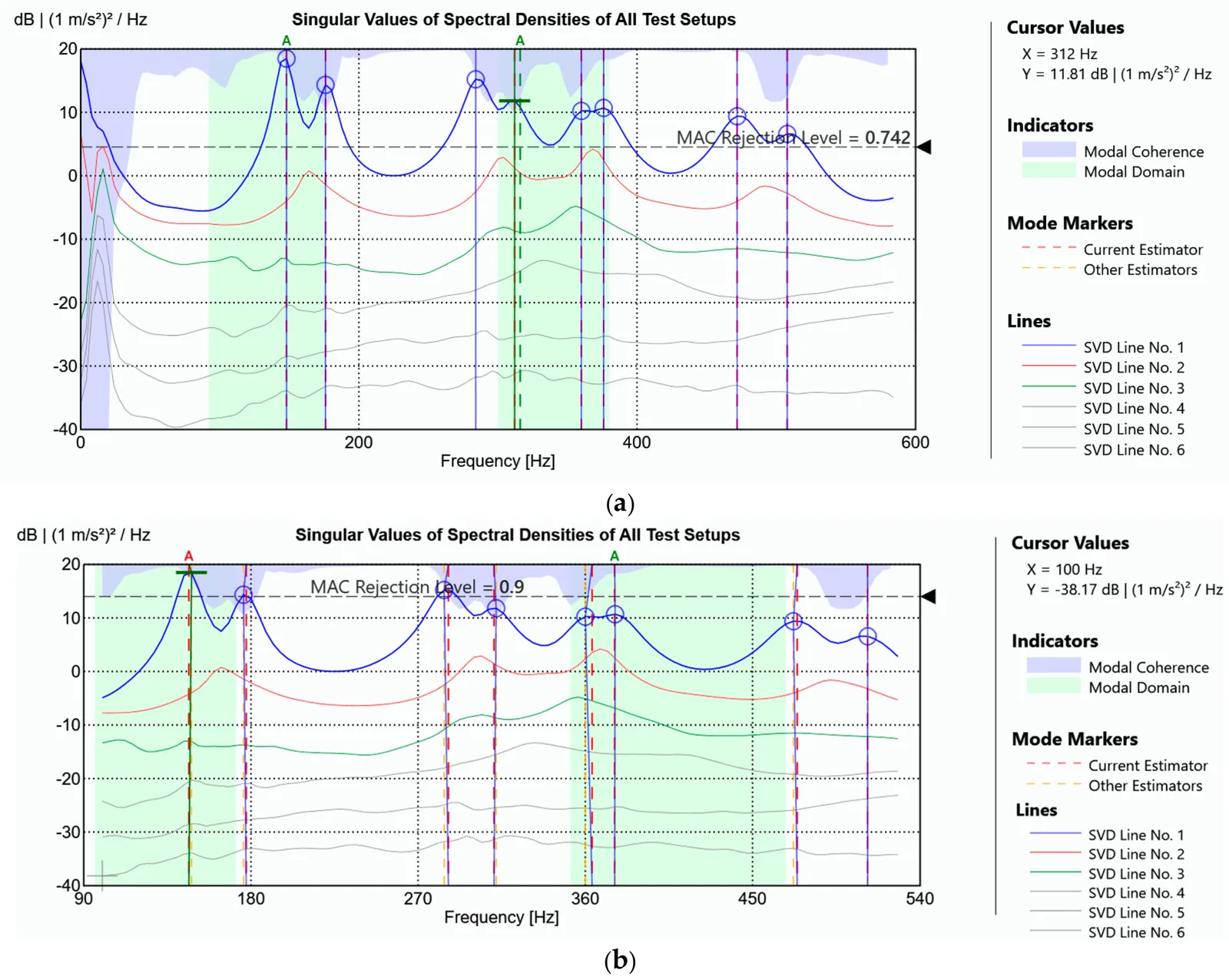
Representative results used for modal parameter identification of the GC = 68% reference specimen: (**a**) Frequency Domain Decomposition (FDD); (**b**) Enhanced Frequency Domain Decomposition (EFDD).

The FDD analysis identified the first six natural frequencies at 148, 176, 284, 312, 376, and 472 Hz. The corresponding EFDD estimates were 146.584, 177.441, 286.282, 310.830, 375.802, and 474.049 Hz, respectively. The close correspondence between the FDD and EFDD frequency estimates supports consistent identification of the same six structural modes using both decomposition procedures. Representative FDD and EFDD identification results are presented in Figure 6.

The EFDD-derived damping ratios for Modes 1–6 were 3.051%, 2.722%, 2.558%, 1.924%, 1.744%, and 2.130%, respectively. Within this reference measurement, damping generally decreased from the first toward the higher-order modes, reaching its minimum for Mode 5 before increasing slightly for Mode 6. Because damping was evaluated only for the GC = 68% reference specimen, these values are reported as modal characteristics of the reference laminate and are not used to establish a relationship between damping and gel content.

**Figure 7:**
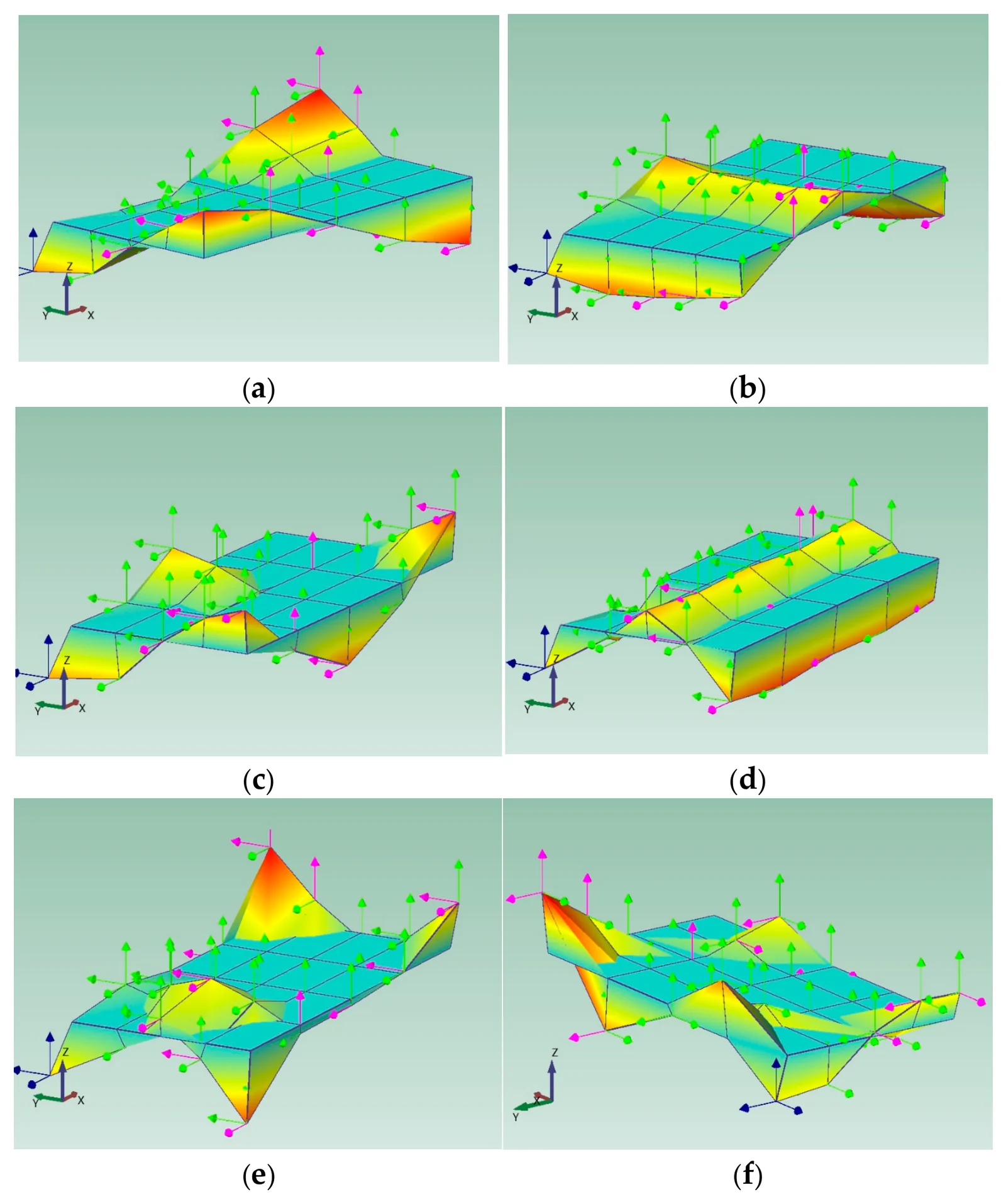
Experimentally identified mode shapes corresponding to the first six elastic vibration modes of the glass–POE–glass laminate: (**a**) Mode 1; (**b**) Mode 2; (**c**) Mode 3; (**d**) Mode 4; (**e**) Mode 5; (**f**) Mode 6.

The experimentally identified deformation patterns corresponding to the first six elastic modes are presented in Figure 7. These mode shapes were subsequently used together with the natural frequencies to establish modal correspondence with the FE results and to maintain consistent mode tracking across the investigated GC levels.

### 3.2. Effect of POE Gel Content on Natural Frequencies

The experimentally identified natural frequencies exhibited a measurable dependence on POE gel content, although the response was not monotonic over the investigated GC range. The FDD natural frequencies obtained for the first six vibration modes at the five GC levels are summarized in Table 4. The reported frequencies represent mean values obtained from five independently manufactured laminate specimens at each GC level, with five repeated modal measurements performed on each specimen. The mean values are presented to the nearest whole hertz. Although independent specimen replication and repeated measurements were performed, the individual frequency estimates are not available in the retained dataset; therefore, standard deviations, confidence intervals, and separate repeatability estimates cannot be calculated retrospectively. Mode 1 showed only a small variation, with mean frequencies between 147 and 149 Hz. In contrast, progressively larger absolute frequency differences were observed for several of the higher-order modes.

The magnitude of the frequency variation depended strongly on the vibration mode. Across the investigated GC range, the peak-to-peak frequency variation was 2 Hz for Mode 1, 7 Hz for Mode 2, 9 Hz for Mode 3, 13 Hz for Mode 4, 17 Hz for Mode 5, and 15 Hz for Mode 6 (Table 5). Relative to the maximum frequency observed for each mode, these variations correspond to approximately 1.34%, 3.83%, 3.07%, 4.00%, 4.33%, and 3.09%, respectively. Thus, Mode 1 exhibited substantially lower sensitivity to changes in curing state than the higher-order modes, while the largest relative variation was observed for Mode 5.

**Table 5:** Frequency variation in the first six vibration modes across the investigated POE gel-content range.

Mode	Maximum Frequency, Hz	Minimum Frequency, Hz	Frequency Range, Hz	Relative Range, %	GC at Minimum Frequency, %
1	149	147	2	1.34	49
2	183	176	7	3.83	68
3	293	284	9	3.07	68
4	325	312	13	4.00	68
5	393	376	17	4.33	68
6	485	470	15	3.09	49

**Figure 8:**
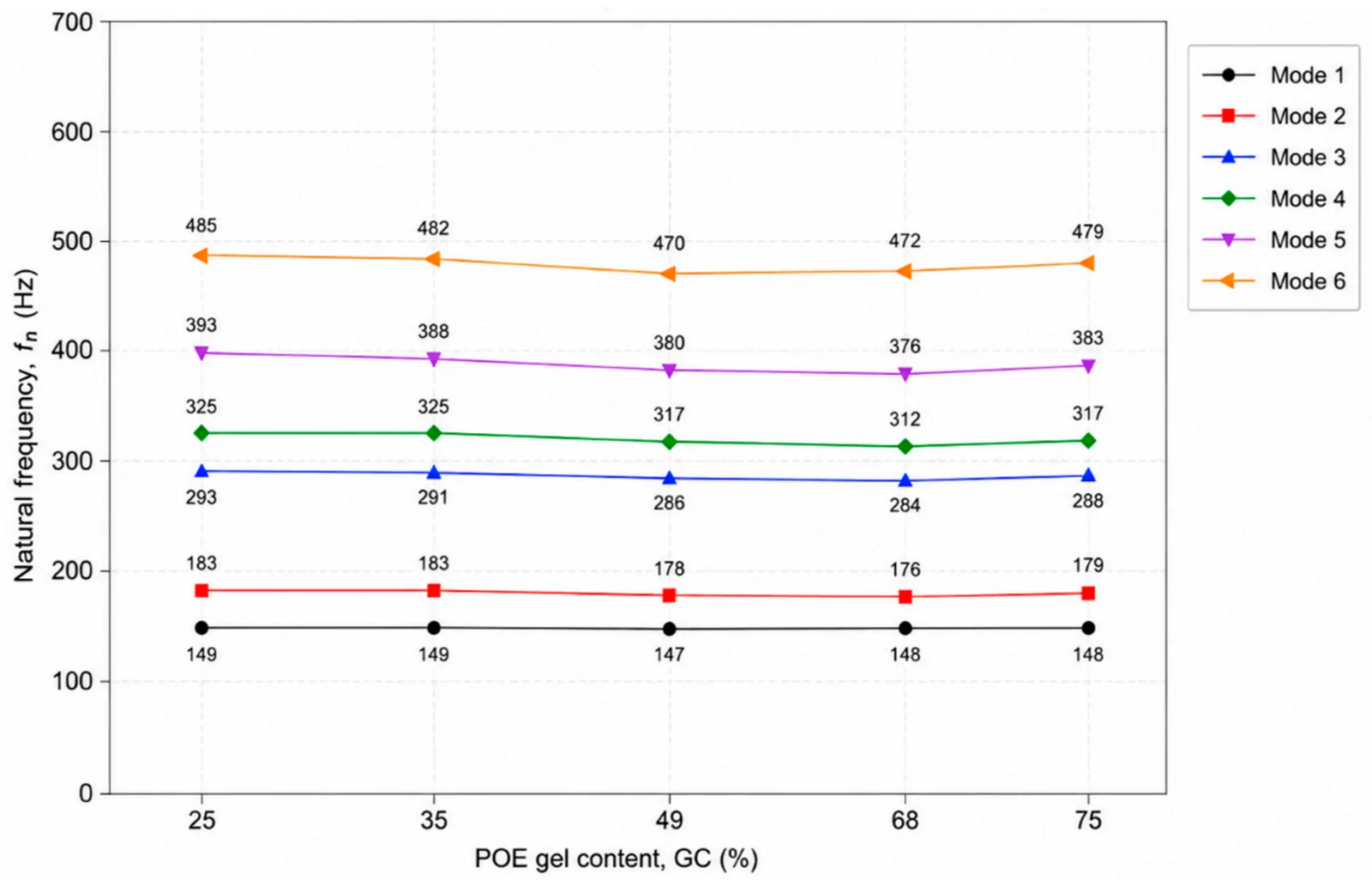
Experimentally identified natural frequencies of the first six vibration modes as a function of POE gel content.

The frequency–GC relationship was not strictly monotonic in the mean modal-frequency values. Modes 2–5 generally exhibited decreasing mean natural frequencies as GC increased from 25% toward 68%, followed by an apparent partial frequency recovery at GC = 75% (Figure 8). For these modes, the lowest mean frequencies were observed at GC = 68%: 176 Hz for Mode 2, 284 Hz for Mode 3, 312 Hz for Mode 4, and 376 Hz for Mode 5. At GC = 75%, the corresponding mean frequencies increased to 179, 288, 317, and 383 Hz, respectively. Mode 6 exhibited a somewhat different pattern, reaching its lowest mean frequency of 470 Hz at GC = 49% before increasing to 472 Hz at GC = 68% and 479 Hz at GC = 75%. Mode 1 remained comparatively insensitive, varying only between 147 and 149 Hz, with its lowest mean frequency also occurring at GC = 49%. Although five independent laminate specimens and five repeated modal measurements per specimen were included at each GC level, the individual frequency estimates are not available for retrospective uncertainty analysis. Consequently, it cannot be established whether the apparent frequency recovery at GC = 75% exceeds specimen-to-specimen and measurement-to-measurement variability. This non-monotonic pattern is therefore reported as a descriptive observation rather than a statistically confirmed curing-related effect.

These results indicate that the sensitivity of the modal response to curing state is mode-dependent. The first mode showed only a small frequency variation over the investigated GC range, whereas Modes 2–6 exhibited relative ranges of approximately 3–4%. Among the investigated modes, Mode 5 showed the largest relative frequency variation (4.33%). The larger frequency variations observed for several higher-order modes suggest that these modes may provide more informative indicators for distinguishing changes in the effective mechanical state of the laminate than the first global mode alone.

### 3.3. Finite Element Comparison for the Reference Laminate

The FE eigenfrequency analysis was compared with the experimentally identified FDD results for the GC = 68% reference specimen. The comparison was performed for the first six elastic vibration modes, with modal correspondence established on the basis of both natural frequencies and the associated deformation patterns. The FE analysis was used to assess whether the simplified numerical representation reproduced the principal modal characteristics of the laminate rather than to predict the experimentally observed dependence of natural frequency on gel content (Table 6).

**Table 6:** Comparison of experimentally identified and FE-predicted natural frequencies for the GC = 68% reference laminate.

Mode	Experimental FDD Frequency, Hz	FE Frequency, Hz	Relative Error, %
1	148	151.70	2.50
2	176	184.87	5.04
3	284	304.06	7.06
4	312	318.72	2.15
5	376	405.42	7.82
6	472	516.54	9.44

The FE-predicted natural frequencies showed generally satisfactory agreement with the experimentally identified FDD frequencies for the GC = 68% reference laminate. The relative differences between the numerical and experimental frequencies ranged from 2.15% to 9.44% across the first six elastic modes. The closest agreement was obtained for Mode 4, for which the experimental and FE frequencies were 312 and 318.72 Hz, respectively, corresponding to a relative difference of 2.15%. The largest difference was observed for Mode 6, with experimental and FE frequencies of 472 and 516.54 Hz, respectively, corresponding to 9.44%.

The FE model systematically overpredicted the experimentally identified natural frequencies for all six modes. The magnitude of the discrepancy generally increased for several higher-order modes, although this trend was not strictly monotonic, as demonstrated by the comparatively small difference obtained for Mode 4. Considering the simplifying assumptions of homogeneous and linear-elastic constituent properties, perfectly bonded glass–POE interfaces, and idealized free–free boundary conditions, the FE results are considered sufficient for qualitative modal correspondence and structural interpretation of the *GC* = 68% reference laminate. However, the FE–experimental frequency differences (2.15–9.44%) are comparable to or larger than the experimentally observed relative frequency variations across the investigated *GC* levels (1.34–4.33%). Therefore, the FE results are not used as quantitative evidence of sensitivity to curing state or as validation of the experimentally observed frequency–*GC* relationship.

**Figure 9:**
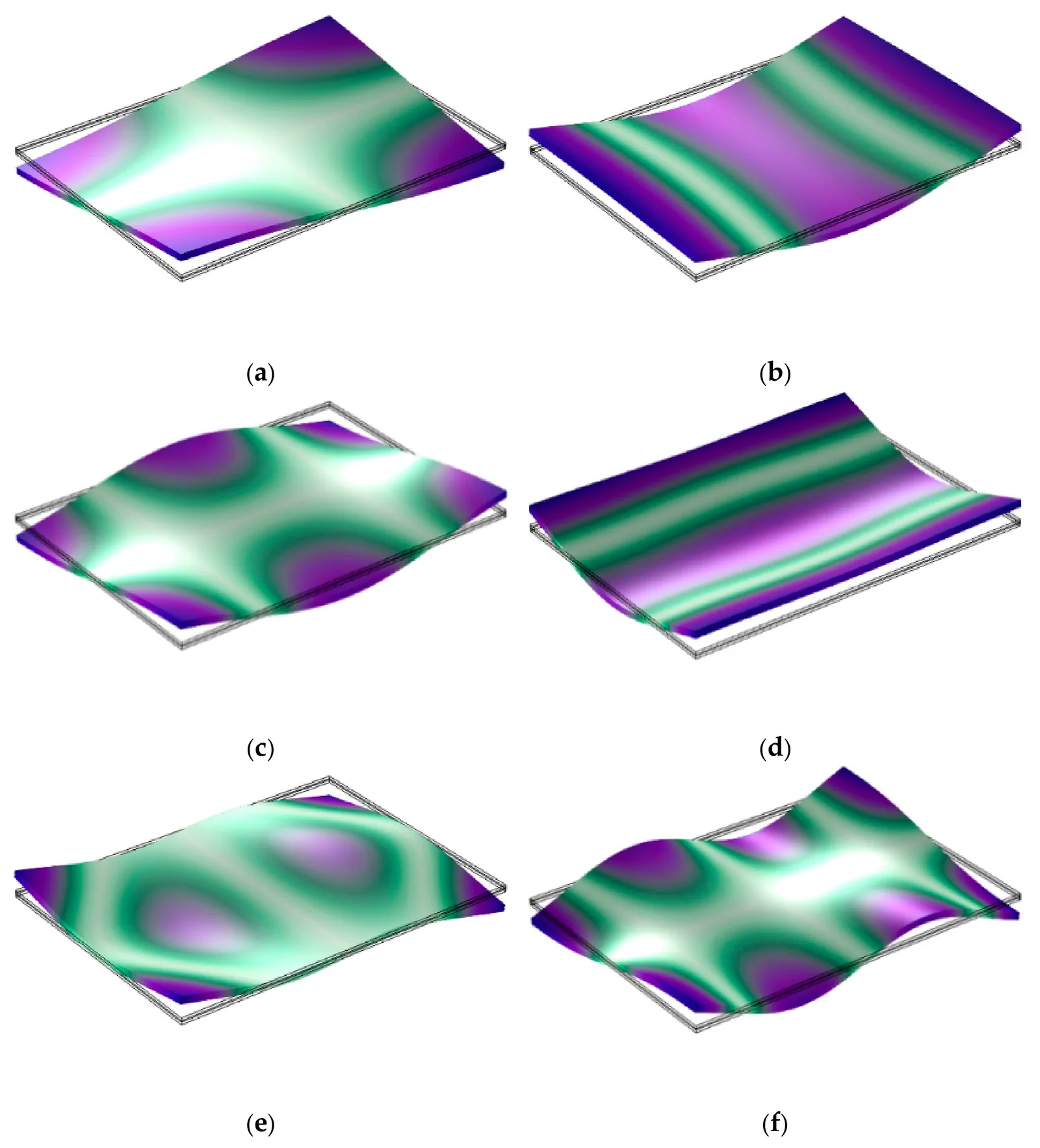
FE-predicted mode shapes for the GC = 68% reference laminate corresponding to the experimentally identified modes shown in Figure 7: (**a**) Mode 1; (**b**) Mode 2; (**c**) Mode 3; (**d**) Mode 4; (**e**) Mode 5; (**f**) Mode 6.

The correspondence between the experimentally identified mode shapes shown in Figure 7 and the FE-predicted deformation patterns is further illustrated in Figure 9.

Overall, the FE deformation patterns reproduced the principal spatial characteristics of the experimentally identified modes, supporting the adopted modal correspondence between the numerical and experimental results. However, the systematic overprediction of the natural frequencies indicates that the simplified linear-elastic FE representation does not fully capture the effective dynamic compliance of the POE-coupled laminate.

### 3.4. Results of the Calibrated Partial-Composite-Action Model

The partial-composite-action model was calibrated using the experimentally identified first natural frequency, as described in Section 2.6. The calibration resulted in a partial-composite-action coefficient η of approximately 0.26–0.27. The corresponding effective bending stiffness, D_eff_, was approximately 1240–1275 N·m, yielding a calculated first natural frequency of approximately 147–149 Hz. This range is consistent with the experimentally identified first-mode frequencies of 147–149 Hz across the investigated GC levels (Table 7).

The calibrated value of η indicates an intermediate structural state between the limiting cases of completely uncoupled glass layers and full composite action. Within the adopted effective-stiffness formulation, approximately 26–27% of the theoretically available increase in bending stiffness between these two limiting states is represented by the calibrated model. The result therefore indicates significant but incomplete mechanical coupling between the glass layers through the POE interlayer. Importantly, η should be interpreted as an effective structural calibration parameter rather than as a direct material property of POE.

**Table 7:** Calibrated parameters and principal results of the partial-composite-action model.

Parameter	Symbol	Calibrated/Model Value
Partial-composite-action coefficient	η	0.26–0.27
Effective bending stiffness	*D_eff_*	1240–1275 N·m
Calculated first natural frequency	*f*_1_	147–149 Hz

The calibrated model reproduces the experimentally observed first-mode frequency by construction because Mode 1 was used as the calibration target. Therefore, agreement for this mode should not be interpreted as independent model validation. In the present study, the analytical model is used primarily to provide an effective-stiffness interpretation of the first-mode response and the degree of partial mechanical coupling between the glass layers, rather than as an independently validated predictor of the complete modal spectrum.

The analytical formulation is intended to represent the global dynamic stiffness of the laminate rather than the detailed dependence of modal frequency on gel content. Accordingly, the calibrated η range should be regarded as representative of the average partial composite interaction under the investigated conditions. The experimentally observed non-monotonic frequency–GC relationship would require a more advanced formulation in which the effective interlayer interaction varies with curing state and, potentially, vibration frequency.

### 3.5. Statistical Analysis Results

The exploratory statistical analysis confirmed that the relationship between POE gel content and natural frequency differed among the investigated vibration modes. Pearson’s correlation coefficients were negative for all six modes, ranging from −0.555 for Mode 1 to −0.861 for Mode 4. Modes 2–5 exhibited comparatively stronger negative linear associations with gel content, whereas Modes 1 and 6 showed weaker linear correlations. These results are consistent with the experimentally observed overall decrease in frequency with increasing GC over much of the investigated range, while also reflecting the non-monotonic frequency recovery observed at the higher GC levels.

Because the experimental frequency–GC relationships were not strictly linear, second-order polynomial regressions were additionally fitted to the data. The quadratic models produced coefficients of determination (R^2^) ranging from 0.654 to 0.906. The highest R^2^ was obtained for Mode 5 (0.906), followed by Mode 3 (0.871) and Mode 6 (0.842). Mode 1 exhibited the lowest R^2^ (0.654), consistent with its comparatively small frequency variation across the investigated GC range. The quadratic fits therefore described the observed trends more closely for several higher-order modes than for the first global mode.

The absolute deviations between the quadratic fits and the experimentally identified frequencies were small relative to the modal-frequency magnitudes. RMSE values ranged from 0.440 Hz for Mode 1 to 2.288 Hz for Mode 6, while MAE values ranged from 0.386 to 2.115 Hz. The statistical results are summarized in Table 8.

Given that only five discrete GC levels were available for each mode, these statistical measures should be interpreted descriptively. In particular, the relatively high R^2^ values obtained for several quadratic fits do not establish a validated predictive relationship between gel content and natural frequency. Rather, they quantify the observed curvature of the present dataset and support the experimental observation that higher-order modal frequencies are generally more responsive to changes in POE curing state than the first mode.

## 4. Discussion

The experimental results support the interpretation that the curing state of the POE interlayer produces measurable changes in the dynamic response of the glass–POE–glass laminate. However, the relationship between gel content and natural frequency was neither uniform across the investigated modes nor strictly monotonic. The first vibration mode varied by only 2 Hz across the complete GC range, whereas the higher-order modes exhibited substantially larger peak-to-peak variations of 7–17 Hz. Modes 2–5 generally decreased in frequency as GC increased from 25% to 68%, followed by a partial recovery at GC = 75%. Mode 6 exhibited a different response, with its minimum frequency occurring at GC = 49%. These observations indicate that gel content cannot be interpreted as a simple scalar parameter producing a proportional change in the global stiffness of the laminate.

A possible physical interpretation of the observed non-monotonic trend is that gel content characterizes the solvent-insoluble fraction of the crosslinked polymer network but does not directly quantify the elastic or shear modulus of the POE interlayer. The structural modal response is governed by the combined effects of constituent properties and mechanical interaction between the glass layers. Changes in curing state could therefore influence effective shear transfer through the POE interlayer without producing a directly proportional change in gel content and laminate bending stiffness. However, the present study did not independently determine curing-dependent POE mechanical properties or quantify the dispersion of the individual modal-frequency measurements. Consequently, the apparent frequency recovery at GC = 75% cannot be conclusively attributed to a change in interlayer shear coupling rather than experimental variability. The proposed mechanical explanation should therefore be regarded as a hypothesis consistent with laminated-structure mechanics, not as a mechanism demonstrated by the present measurements.

The greater frequency variation observed for the higher-order modes suggests that they provide greater sensitivity to curing-induced changes in the mechanical state of the interlayer than the first global mode. Higher-order deformation patterns can involve increased spatial variation in curvature and therefore provide multiple modal indicators of changes in the effective laminate stiffness. This finding is also supported by the exploratory statistical analysis, in which several higher-order modes exhibited stronger associations between GC and natural frequency than Mode 1. Nevertheless, the response was mode-dependent, as demonstrated particularly by the different behavior of Mode 6. A multi-mode assessment is therefore preferable to reliance on a single natural frequency when modal characterization is considered for curing-state screening.

The modal identification results presented in Figure 6 and Figure 7 provide additional structural context for this interpretation. The close correspondence between the FDD and EFDD frequency estimates for the GC = 68% reference specimen indicates consistent identification of the same modal components using the two decomposition procedures, while the experimentally identified mode shapes support the interpretation that the evaluated spectral peaks correspond to distinct elastic deformation patterns rather than to frequency peaks considered in isolation. This distinction is important for layered structures because previous experimental studies of laminated glass have shown that polymer interlayers influence both natural frequencies and damping characteristics [21,22,23]. Furthermore, recent modal investigations of photovoltaic modules have demonstrated that changes in module construction and in the mechanical contribution of individual layers are reflected in their modal characteristics [26]. In this context, Figure 6 and Figure 7 support the use of a mode-specific rather than purely frequency-based interpretation of the present results: the observed GC-related frequency changes are evaluated for consistently tracked structural modes whose response is governed by the mechanical interaction of the laminate constituents.

The FE analysis provided a complementary numerical reference for interpretation of the experimentally identified modal characteristics of the GC = 68% laminate. The calculated deformation patterns reproduced the principal spatial characteristics of the experimentally identified modes, supporting the adopted correspondence between the experimental and numerical modal sequences. The FE model nevertheless systematically overpredicted the measured natural frequencies, with relative differences ranging from 2.15% to 9.44%. This discrepancy is consistent with the simplifying assumptions adopted in the numerical representation, including homogeneous and linear-elastic constituent properties, perfectly bonded glass–POE interfaces, and idealized free–free boundary conditions. In particular, representation of the POE interlayer by a single effective elastic modulus cannot reproduce its potentially frequency-dependent and curing-dependent mechanical response. The FE results should therefore be interpreted primarily as support for modal correspondence and structural interpretation rather than as a validated constitutive model of the POE interlayer. Importantly, the FE–experimental frequency differences of 2.15–9.44% are comparable to or larger than the experimentally observed relative frequency variations of 1.34–4.33% across the investigated GC levels. Consequently, the FE analysis cannot be regarded as quantitative validation of the observed curing-state sensitivity or of the frequency–GC relationship. The mode-dependent FE discrepancies may themselves partly reflect the simplifying assumptions of the numerical model, particularly the neglect of the frequency-, temperature-, and curing-dependent viscoelastic behavior of the POE interlayer and the idealization of the experimental support conditions.

The calibrated partial-composite-action model provides a complementary mechanical interpretation of these observations. The identified coefficient η ≈ 0.26–0.27 places the laminate between the limiting conditions of completely uncoupled glass layers and full composite action, indicating substantial but incomplete mechanical interaction through the POE interlayer. The corresponding effective bending stiffness of approximately 1240–1275 N·m reproduces the experimentally observed first-mode frequency range of approximately 147–149 Hz. Because η was calibrated using the first-mode response, this agreement does not constitute independent model validation. Instead, the model demonstrates that the measured global dynamic response can be represented consistently by an intermediate effective-stiffness state, supporting the interpretation of the POE layer as a compliant mechanical coupling medium between the two glass layers.

From a practical perspective, the present results indicate that modal characterization may provide a useful non-destructive screening tool for assessing changes in the curing state of POE-containing laminated structures. Unlike solvent-extraction methods, vibration-based measurements do not require removal or destruction of the encapsulant and characterize the integrated mechanical response of the laminated specimen. The observed differences among the higher-order modal frequencies suggest that a multi-mode frequency signature could potentially be used to distinguish laminates with different curing states. However, because the frequency–GC relationship was non-monotonic, the present results do not support determination of an unknown gel-content value from a single measured natural frequency. The method should therefore presently be regarded as a comparative screening approach rather than as a quantitative replacement for conventional gel-content determination.

In comparison with conventional encapsulant-characterization methods, the principal potential advantage of modal characterization is that it evaluates the integrated mechanical response of the laminated structure without requiring extraction of the polymer interlayer. Solvent-extraction measurements provide a direct operational measure of the insoluble gel fraction, while calorimetric, dynamic-mechanical, rheological, and spectroscopic methods provide material-level information on curing reactions, viscoelastic behavior, or chemical transformations [9,12,24]. These methods therefore remain more appropriate when direct characterization of the encapsulant material or quantitative determination of curing-related material properties is required. By contrast, vibration-based characterization is sensitive to the combined structural effects of constituent properties and interlayer mechanical coupling, which may make it suitable for rapid comparative screening of laminated components without destructive sampling. This structural-level sensitivity is consistent with previous modal investigations of laminated glass and photovoltaic modules, where changes in interlayer properties or module construction were reflected in the measured modal response [21,22,23,26]. However, the same integrated sensitivity also represents an important limitation: modal parameters can be influenced not only by the curing state of the encapsulant but also by specimen geometry, boundary conditions, temperature, constituent properties, interfaces, and additional module components. Consequently, the present method should be regarded as complementary to, rather than a replacement for, established material-level characterization techniques until these confounding influences are quantified and the approach is validated on complete photovoltaic modules.

Several limitations of the present study should be considered when interpreting the results. First, although five independently manufactured laminate specimens were tested at each GC level, only five discrete gel-content levels were investigated, which limits the statistical resolution of the observed frequency–GC relationships and precludes establishment of a generalized predictive model. Furthermore, while five repeated modal measurements were performed on each specimen and the frequencies reported in Table 4 represent mean values, the individual specimen-level and measurement-level frequency estimates are not available in the retained dataset. Consequently, standard deviations, confidence intervals, and quantitative estimates of specimen-to-specimen and measurement-to-measurement variability cannot be reconstructed retrospectively. The GC values were determined as the means of five solvent-extraction determinations performed on separate PTFE-containing preparations processed in the same lamination cycles as the corresponding modal-test specimens. However, the individual GC determination results are not available for retrospective calculation of their dispersion. Moreover, although the PTFE-containing preparations and modal-test laminates were subjected to the same processing conditions, the equivalence of their resulting POE gel contents was not independently verified. Therefore, the assigned GC values should be interpreted as curing-condition descriptors rather than as direct measurements of the gel content within each modal-test specimen. Second, the investigated specimens represented a simplified glass–POE–glass configuration without photovoltaic cells. This configuration was intentionally selected to isolate the mechanical contribution of the POE interlayer, but the dynamic response of complete photovoltaic modules may additionally be affected by cells, metallization, interconnects, local interfaces, frames, junction boxes, and other structural components. These additional structural features may alter modal frequencies and mode shapes and may be particularly relevant to higher-order modes; consequently, the sensitivity of these modes to POE curing state observed in the simplified laminates cannot yet be assumed to transfer directly to complete photovoltaic modules. Third, damping was evaluated only for the GC = 68% reference specimen and therefore cannot presently be used to assess curing-state dependence. In addition, the separate contributions of impact-to-impact frequency variability, support-condition variability, and sensor mounting/repositioning effects were not quantified. Because the observed GC-related frequency differences are relatively small, future studies should retain individual measurement results, quantify these sources of variability, and compare the resulting uncertainty estimates directly with the magnitude of the curing-related modal shifts. Finally, the FE and analytical models employ simplified representations of the POE interlayer and do not explicitly account for its complete viscoelastic, frequency-dependent, temperature-dependent, or curing-dependent constitutive behavior.

Future work should therefore focus on expanding the experimental dataset to include a larger number of specimens and additional curing states, with independent specimens used for calibration and validation. Particular attention should be given to the higher-order modes identified here as more sensitive to changes in curing state and to the development of multi-modal classification approaches rather than single-frequency correlations. Measurements on complete photovoltaic laminates containing cells will also be required to determine whether the observed modal indicators remain sufficiently sensitive under more representative structural conditions. In parallel, characterization of the curing-dependent and frequency-dependent mechanical properties of POE would enable development of more physically based numerical models linking polymer state, interlayer shear transfer, and laminate modal response. Such validation is necessary before vibration-based characterization can be used for quantitative estimation of unknown POE gel content or transferred to industrial quality-control applications.

## 5. Conclusions

This study investigated the feasibility of using vibration-based modal characterization as a non-destructive indicator of the curing state of POE interlayers in simplified glass–POE–glass laminate specimens relevant to photovoltaic module structures. Five experimentally determined gel-content levels, ranging from 25% to 75%, were investigated using FDD and EFDD, and the first six elastic vibration modes were identified. The mean natural frequencies exhibited mode-dependent differences across the investigated curing conditions, suggesting that the modal response of the laminated structure may be sensitive to the mechanical state of the POE interlayer. However, the observed frequency–GC relationship was not strictly monotonic, and the absence of retained individual frequency estimates prevents statistical confirmation that these differences exceed experimental variability.

The magnitude of the observed variation in mean natural frequencies was strongly mode-dependent. Mode 1 varied by only 2 Hz across the investigated GC range, whereas Modes 2–6 exhibited peak-to-peak variations of 7–17 Hz, corresponding to relative ranges of approximately 3–4.3%. Modes 2–5 generally decreased in mean frequency as GC increased from 25% to 68%, followed by an apparent partial recovery at 75% GC. Among the investigated modes, Mode 5 exhibited the largest relative frequency variation of 4.33%. These descriptive results suggest that a multi-mode frequency assessment, particularly including higher-order modes, may provide more information about differences between the investigated curing conditions than the first global mode alone. However, the absence of individual frequency estimates and corresponding dispersion measures prevents confirmation that the observed differences, including the apparent recovery at GC = 75%, exceed experimental variability.

The numerical and analytical models provided complementary structural interpretation of the experimental observations. The FE model reproduced the principal experimentally identified deformation patterns, while the calculated natural frequencies differed from the measured values by 2.15–9.44%. The calibrated partial-composite-action model yielded η ≈ 0.26–0.27 and an effective bending stiffness of approximately 1240–1275 N·m, indicating an intermediate structural condition between uncoupled glass layers and full composite action. These results support the interpretation that the POE interlayer provides significant but incomplete mechanical coupling between the glass layers.

The present findings support modal characterization as a potentially rapid and non-destructive screening approach for distinguishing changes in POE curing state. However, the limited number of investigated GC levels, the simplified glass–POE–glass specimens without photovoltaic cells, and the non-monotonic frequency–GC relationship currently preclude quantitative estimation of unknown gel content from modal frequencies alone. Validation using larger independent specimen sets, complete photovoltaic laminates, and curing- and frequency-dependent POE material characterization is required before the approach can be developed into a quantitative industrial quality-control method.

## Figures and Tables

**Table 1:** Nominal geometrical and material properties of the glass–POE–glass laminate used in the structural models.

Layer	Thickness, mm	Density, kg/m^3^	Young’s Modulus, MPa	Poisson’s Ratio
Glass layer 1	3.6	2500	70,000	0.23
POE interlayer	0.8	857	11 *	0.45
Glass layer 2	3.6	2500	70,000	0.23

* The POE Young’s modulus listed in Table 1 represents the effective value adopted in the numerical model of the GC = 68% reference specimen and should not be interpreted as an experimentally measured modulus applicable to all investigated gel-content levels.

**Table 2:** Geometrical and material parameters adopted in the analytical model.

Parameter	Symbol	Value
Specimen length	a	0.415 m
Specimen width	b	0.298 m
Aspect ratio	a/b	1.393
Total laminate thickness	h	8.0 mm
Total glass thickness	h_g,total_	7.2 mm
Individual glass thickness	h_g_	3.6 mm
POE interlayer thickness	h_POE_	0.8 mm
Glass Young’s modulus	E_g_	70 GPa
Glass Poisson’s ratio	ν_g_	0.23
Glass density	ρ_g_	2500 kg/m^3^
POE Young’s modulus	E_POE_	3–12 MPa
POE Poisson’s ratio	ν_POE_	0.45
POE density	ρ_POE_	857 kg/m^3^

**Table 3:** Modal parameters identified using FDD and EFDD for the GC = 68% reference specimen.

Mode	FDD Frequency, Hz	FDD Complexity, %	EFDD Frequency, Hz	EFDD Damping Ratio, %	EFDD Complexity, %
1	148	0.455	146.584	3.051	0.426
2	176	0.327	177.441	2.722	0.337
3	284	1.061	286.282	2.558	1.238
4	312	4.040	310.830	1.924	4.614
5	376	6.633	375.802	1.744	3.983
6	472	3.709	474.049	2.130	3.975

**Table 4:** Mean experimentally identified FDD natural frequencies for the investigated POE gel-content levels (five independent laminate specimens per GC level; five repeated measurements per specimen).

Mode	GC 25%	GC 35%	GC 49%	GC 68%	GC 75%
1	149	149	147	148	148
2	183	183	178	176	179
3	293	291	286	284	288
4	325	325	317	312	317
5	393	388	380	376	383
6	485	482	470	472	479

**Table 8:** Descriptive statistical parameters characterizing the relationship between POE gel content and experimentally identified natural frequency.

Mode	Pearson’s r	R^2^	RMSE, Hz	MAE, Hz
1	−0.555	0.654	0.440	0.386
2	−0.812	0.788	1.284	1.197
3	−0.778	0.871	1.170	1.033
4	−0.861	0.812	2.201	2.014
5	−0.795	0.906	1.832	1.612
6	−0.567	0.842	2.288	2.115

## Data Availability

The raw data supporting the conclusions of this article will be made available by the authors on request.
